# Pragmatic Strategy for Fecal Specimen Storage and the Corresponding Test Methods for *Clostridioides difficile* Diagnosis

**DOI:** 10.3390/pathogens10081049

**Published:** 2021-08-18

**Authors:** Seong Won Nho, Minjae Kim, Seong-Jae Kim, Steven L. Foley, Rajesh Nayak, Ohgew Kweon, Carl E. Cerniglia

**Affiliations:** 1 Division of Microbiology, National Center for Toxicological Research, U.S. Food and Drug Administration, Jefferson, AR 72079, USA; seongwon.nho@fda.hhs.gov (S.W.N.); minjaekim45@gmail.com (M.K.); seongjae.kim@fda.hhs.gov (S.-J.K.); steven.foley@fda.hhs.gov (S.L.F.); 2 Office of Regulatory Compliance and Risk Management, National Center for Toxicological Research, U.S. Food and Drug Administration, Jefferson, AR 72079, USA; Rajesh.nayak@fda.hhs.gov

**Keywords:** *Clostridioides difficile*, CDI diagnosis, fecal specimen, real-time PCR, storage condition, bibliomic data

## Abstract

The quality of fecal specimens is one of the factors responsible for successful *Clostridioides difficile* infection (CDI) diagnosis. The quality depends largely on the storage conditions, including the temperature and time period. In this study, we organized the outputs of previous studies, filled experimental gaps in the knowledge of storage conditions, and introduced a pragmatic strategy for fecal storage for CDI diagnosis. A 5-step pathway was adopted to develop the fecal specimen storage strategy as follows: step 1, bibliomic analysis; step 2, experimental gap-filling; step 3, comparative evaluation; step 4, strategy development; step 5, internal review. Step 1 identified eight articles providing experimental information on the effects of fecal specimen storage conditions on the effectiveness of *C. difficile* detection methods. Step 2 provided additional quantitative data on *C. difficile* vegetative and spore cell viability and DNA stability. All previous and current results were compared (step 3). In step 4, fir general and nine special strategies were developed, followed by an internal review of the overall approaches (step 5). It is recommended to separate fecal samples into aliquots before testing and storing them. It is particularly recommended that fecal specimen samples be stored for CDI diagnosis at 4 °C for up to 60 days for all test methods.

## 1. Introduction

A Gram-positive, spore-forming, and obligate anaerobic bacterium, *Clostridioides difficile*, is responsible for the majority of recently increasing cases of infectious antibiotic-associated diarrhea and pseudomembranous colitis [1,2,3]. *C. difficile* infection (CDI) is a major medical problem in many health care facilities, including hospitals, long-term care facilities, and nursing homes [4,5,6]. Accurate and timely diagnosis is necessary both for appropriate clinical management of the patients and for the timely implementation of infection control [7,8].

Traditionally, a cell culture cytotoxicity assay has been widely used for CDI diagnosis due to its high sensitivity, together with toxigenic culture [9,10]. An enzyme immunoassay (EIA) for toxins A/B and glutamate dehydrogenase (GDH) has also been one of the widely used test methods until recently, although it has a low sensitivity and specificity compared to toxigenic culture [11,12]. In recent years, several nucleic acid amplification tests (NAATs) based on real-time PCR or loop-mediated isothermal amplification (LAMP) have been developed for the diagnosis of CDI, which directly detect the *tcdA* and *tcdB* genes from stool specimens with high sensitivity (>90%) and specificity (>99%) [13,14]. Many laboratories use stand-alone tests or diagnostic algorithms to aid in the diagnosis of CDI [15]. While each of these test methods or diagnostic algorithms have their own benefits, the most critical factor to the accurate diagnosis of *C. difficile* is the quality of the fecal samples (and the corresponding targets of the test methods, e.g., cell viability, cytotoxicity, DNA stability, etc.). Ideally, it is best to do diagnostic assays immediately after sampling of specimens. Unless properly handled, the quality of a stool sample decreases from the time of collection until testing. Appropriate storage of fecal samples is essential to avoid the introduction of post-collection bias in test result. Several studies have investigated the impact of different storage conditions on the quality of stool samples [16,17,18,19,20,21,22,23]; however, these previous studies have some limitations that minimize their utility and there were experimental gaps in the storage conditions tested in the studies, preventing pragmatic storage strategies from being generated for stand-alone tests or currently accepted CDI diagnosis algorithms.

The aim of this study was to provide a practical handling and storage strategy for fecal samples with regards to the test methods used to aid in the diagnosis of CDI. We conducted a comprehensive review of the published articles (i.e., bibliomic data) in order to systemically organize the storage conditions and of the CDI test methods and results in order to find data gaps. We examined the differences in the numbers of *C. difficile* vegetative and spore cells during storage at −70 °C, −20 °C, 4 °C, and RT over 28 days, which are typical storage temperature conditions. We also examined the stability of *C. difficile* DNA in the fecal sample over the course of sample storage, using a qPCR method that detects *C. difficile* toxin A and B genes. Comparative integration of previous and current results allowed us to update and recommend more pragmatic protocols for fecal handling and storage processes.

## 2. Results

**Figure 1:**
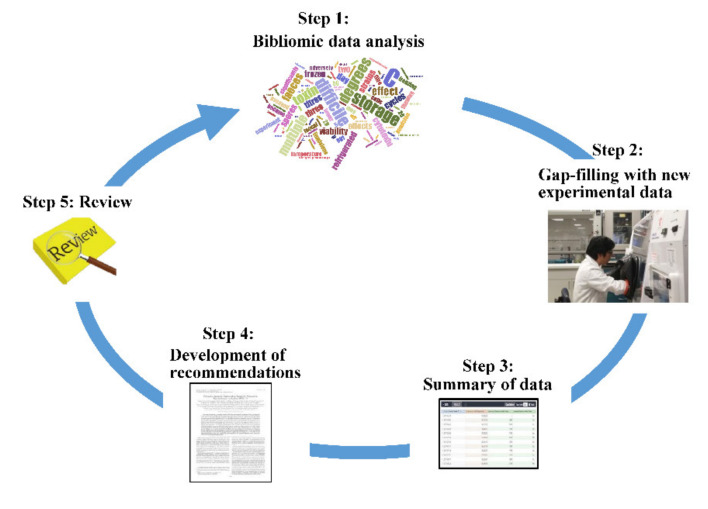
Procedures used to develop practical strategy for fecal specimen handling and storage and CDI detection methods.

Figure 1 shows the procedures used for the development of strategy adopted in this study: step 1, analyze bibliomic data; step 2, perform an experiment to fix the shortfall; step 3, compare and summarize the storage effects; step 4, develop a strategy; step 5, verify the strategy via internal review.

### 2.1. Step 1: Analyze Bibliomic Data 

Out of 13,100 peer reviewed articles retrieved from a primary search using “*C. difficile* [title]”, 8 publications were identified to provide experimental information on the storage effects on the targets of *C. difficile* detection methods. These 8 articles, which had an average number of 36 citations per paper, provided a total of 16 detection method categories (Table 1). Among these, cells, spores, and proteins were used as the targets of *C. difficile* detection in 7 and 6 articles, respectively, while only one article used DNA for the target of *C. difficile* detection (Table 1). The test method, i.e., toxigenic culture, was used in 7 articles, which had 372 citations.

### 2.2. Step 2: Perform an Experiment to Bridge the Gaps

#### 2.2.1. Vegetative Cell and Spore Counts of *C. difficile* at Different Storage Conditions

**Figure 2:**
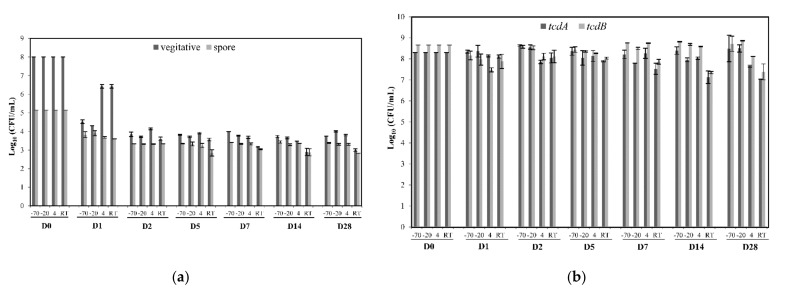
Effects of storage conditions on fecal samples at four different temperatures, namely −70 °C, −20 °C, 4 °C, and RT, for 28 days using stool samples spiked with *C. difficile* ATCC BAA-2155. Numbers of vegetative cells and spores based on plate counting (**a**) and qPCR assay (**b**).

The numbers of viable *C. difficile* cells were measured for all storage conditions. Fecal samples were initially spiked with 8 log CFU of *C. difficile* ATCC BAA-2155 per mL. As shown in Figure 2a, the results of plate counting on day 1 showed that the numbers of vegetative cells markedly decreased by 56.3% and 53.8% compared with the initial cell numbers at temperatures of −70 °C and −20 °C, respectively. At day 2, there were additional decreases in the numbers of viable cells (47.5% and 46.3% of day 0 counts, respectively) at these temperatures. The survival of *C. difficile* vegetative cells stored at 4 °C and RT, on the other hand, showed less of a decrease at day 1 (80% of day 0 counts), in contrast to the counts observed under freezing temperatures. From day 1 until day 28, the numbers of viable cells remained quite constant at 3 storage temperatures −70 °C, −20 °C, and 4 °C, with the exception of the viability from day 2 to day 5 at 4 °C, which was slightly higher than the viability at −70 °C and −20 °C. Over the entire 28 days, among the 4 storage temperatures, the biggest decrease in the viability of vegetative cells was observed at RT. These counts decreased to 45% and to 36.3% at day 2 and day 28, respectively.

Plate counting of the fecal samples treated with alcohol detected gradual decreases in the numbers of *C. difficile* spores for all storage conditions (Figure 2a). The extent of the decreases in the numbers of spores for storage temperatures overall showed similar patterns over 28 days for the vegetative cells. The results of the culture showed that the numbers of spores at storage temperatures of −70 °C, −20 °C, and 4 °C decreased to around 75% at day 1 and around 65% at day 28; however, storage of the below freezing temperatures (−70 °C and −20 °C) appeared to produce slightly higher numbers of spores overall than at 4 °C. The lowest numbers of spores were detected at RT, particularly after day 7.

#### 2.2.2. *C. difficile* DNA Stability at Different Storage Conditions 

The results of the *tcdA* qPCR assay (Figure 2b) showed that when the total *C. difficile* concentrations were estimated from Cq values, the overall concentrations were similar, at 7.8 to 8.6 log CFU per mL, for all time points, except that there were slight decreases in concentrations at 4 °C and RT that dropped to as low as 7.0 to 7.6 log CFU per mL after day 7. The qPCR assay for *tcdB* (Figure 2b) also showed that overall, a similar number of *C. difficile* cells ranged between 8.1 to 8.6 log CFU per mL for all temperatures over time, except a lower number of cells (1.2 log CFU per mL) calculated at RT after day 28, compared with day 0.

### 2.3. Step 3: Compare the Storage Effects 

A total of 9 studies (8 previous studies and this study) were systematically integrated to compare the storage effects on the quality of fecal specimens with *C. difficile* detection (Table 2).

### 2.4. Step 4: Develop Handling and Storage Strategy 

**Figure 3:**
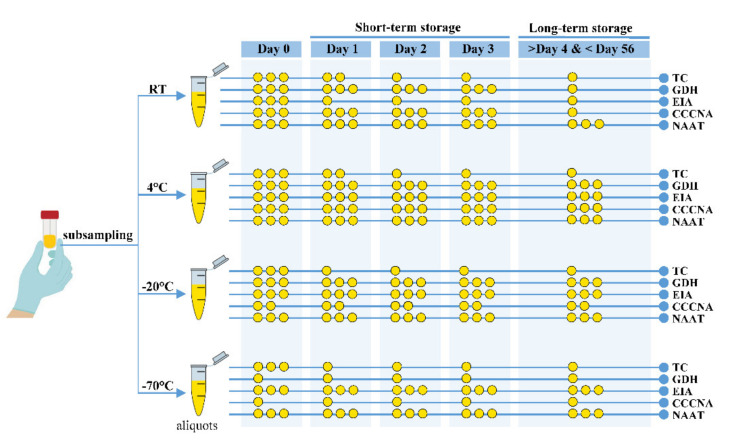
A schematic overview of the practical strategy used for stool specimen handling, storage conditions (temperatures and periods), and the corresponding detection methods for *C. difficile* diagnosis. Circles in yellow represent the relative effectiveness of the strategy based on all experimental information compared in this study. The use of three circles indicates strongly recommended, as rated when the results of *C. difficile* diagnosis were stable under given storage temperature, period, and test methods.

Figure 3 shows the strategy developed for stool sample handling and storage and the corresponding test methods.

The general strategy for fecal sample storage for CDI diagnosis methods is as follows:

- Shorten the handling time;
- Avoid repeated dramatic temperature fluctuation;
- Avoid freeze–thaw cycles;
- Before testing the samples, distribute the feces into aliquots for future application;
- Store the aliquots at room, refrigeration (4 °C), and freezing (−20 °C or −70 °C) temperatures (if possible).

Specific strategies for fecal sample storage for CDI diagnosis methods are as follows:

- At day 0, use stool samples stored at RT or refrigeration temperature (4 °C) for all test methods (TC, GDH, EIA, CCCNA, and NAAT);
- For TC before day 2, use stool samples stored at RT or 4 °C;
- For TC after day 2, use stool samples stored at 4 °C or a freeze temperature of −20 °C or −70 °C;
- For short-term (72 h) GDH assays, use stool samples stored at RT, 4 °C, or −20 °C;
- For long-term (after 72 h) GDH assays, use stool samples stored at 4 °C or −20 °C;
- For EIA during either short-term or long-term storage, use stool samples stored at 4 °C or a freeze temperature of −20 °C or −70 °C;
- For CCCNA during short-term storage, use stool samples stored at RT and 4 °C;
- For CCCNA during long-term storage, use stool samples stored at 4 °C;
- For NAAT, use any stool sample stored at any temperature (RT, 4 °C, −20 °C, or
−70 °C).

### 2.5. Step 5: Verify the Strategy by Internal Review 

Drs. Huizhong Chen and Kidon Sung (within the agency) internally reviewed the strategy.

## 3. Discussion

Collecting and storing fecal specimens for testing is a routine but critical process to ensure accurate results in the clinical laboratory. Stool specimens for CDI diagnosis should be transported to the laboratory as soon as possible. If testing cannot be performed immediately, it is currently recommended that stool specimens be stored at 2 °C to 8 °C for up to 24 hours (or at 4 °C prior to testing) or frozen at −70 °C for longer storage [24]. When toxin testing has been completed, the fecal sample should be frozen at −20 °C for up to 3 months in order to allow culture at a later time for typing if required [25]. The current recommendations cover some of the key steps in handling stool samples; however, neither of these recommendations provides any information on the impacts of more prolonged stool storage at different temperatures on the stability of the test targets. As shown in the integrated data (Table 2), sample storage at different conditions apparently influences the stability of the targets (i.e., bacterium or spore, glutamate dehydrogenase, toxins, and toxin genes) of *C. difficile* test methods with different degrees of influence. It is evident that improperly stored samples can compromise the function of diagnostic methods and can produce misleading results. In this respect, the strategy used in this study, which established a reasonable link between stool storage and the corresponding CDI test methods, can reduce false-negative diagnosis.

Currently, storing fecal specimens at refrigeration temperatures (2–8 °C) is a common practice for short-term storage (up to 24 h or prior to testing) [24]. Interestingly, as revealed in this study, storage at refrigeration temperature (4 °C) of stool specimens used for CDI diagnosis is recommended not only for short-term storage (within 3 days), but also for long-term (~60 days) storage for all the test methods, including TC. Although the numbers of vegetative and spore cells during storage at all temperatures decreased (decreased until 3 days and then stabilized, as shown in Figure 2A until 28 days), previous studies reported that patient stools stored at 4 °C showed consistent binary test results from TC tests over at least 56 days [17,20]. Freeman et al. confirmed that single and multiple exposures of samples to 4 °C had little effect upon the *C. difficile* toxin titer and recommended that specimens should be stored at 4 °C instead of −20 °C to minimize toxin degradation [20]. Using either CDI patient stool samples or contrived fecal samples spiked with *C. difficile* stored at 4 °C, previous studies showed that enzyme immunoassay tests for toxins A/B and GDH also gave very stable test results at 28–120 days (>90% reproducibility) [22]. For molecular assays, as shown in our results, stool samples stored at 4 °C were stable for NAAT for at least 28 days. Storing fecal samples at 4 °C, which is more reliable than subzero storage systems, also provides collateral benefits, such as avoiding repetitive freeze–thaw cycles and reducing storage costs.

Laboratory diagnosis is a crucial part of the management of patients with suspected CDI. A plethora of testing methods have spawned diverse approaches to CDI diagnosis, including 2-step and 3-step testing algorithms and the use of stand-alone tests. Together with the evolution of test methods, *C. difficile* guidelines are also evolving to recommend updated treatments and protocols. Considering that quality of fecal samples is the key to successful diagnosis of CDI, *C. difficile* guidelines should include pragmatic advice on the impacts that stool quality have on the diagnostic approaches used over various storage conditions. A reasonable review process, involving systematic review of the bibliomic data and gap-filling with additional experimental data, is essential to enhance the quality of the recommendations with the aim of updating guidelines. The strategy developed in this study should be continuously updated with new basic and clinical data to ensure the validity of the strategy.

## 4. Materials and Methods

### 4.1. Literature Search

To identify potentially relevant articles, we searched Google Scholar (https://scholar.google.com/), PubMed (https://www.ncbi.nlm.nih.gov/pubmed/), and Web of Science (https://apps.webofknowledge.com/WOS_GeneralSearch_input.do?product=WOS&search_mode=GeneralSearch&SID=5A2X9WMkPLLdOfCjTRE&preferencesSaved=) using the following search terms: “*C. difficile*” in title, “*C. difficile*” in title, AND “storage” in any fields.

### 4.2. Bacterial Culture 

Twenty-one *C. difficile* reference strains (Table A1) were purchased from American Type Culture Collection (ATCC). Cells of *C. difficile* strains were cultured onto cycloserine–cefoxitin–fructose agar (CCFA, OXOID, Cheshire, UK) plates, supplemented with 5% defibrinated horse blood (OXOID), then incubated at 37 °C for 48 h in an AS-580 anaerobic chamber (Anaerobe System, CA, USA). The growth of *C. difficile* was identified on the basis of typical odor and colony morphology. For the storage condition experiments, bacteria anaerobically grown on brain–heart infusion (BHI) agar plates at 37 °C for 48 h were transferred into BHI broth supplemented with 0.5% yeast extract and 0.1% cysteine, then incubated at 37 °C overnight. Cultures were harvested by centrifugation at 13,000 g for 10 min, then subsequently the supernatant was removed.

### 4.3. Spiked Fecal and Storage Conditions

The use of human fecal samples was approved by the FDA Research Involving Human Subjects Committee (RIHSC #16-032T). Fecal samples were obtained from 3 healthy adult individuals and autoclaved to eliminate potential viable *C. difficile* cells and spores to ensure accurate counts of *C. difficile* in subsequent experiments. Autoclaved fecal samples were diluted to 3% (*w*/*v*) with pre-reduced phosphate-buffered saline (PBS, composed of 137 mM NaCl, 2.7 mM KCl, 10 mM Na2HPO4, 2.0 mM KH2PO4, pH 7.4) and spiked with the same volume of BHI broth-cultured *C. difficile* ATCC BAA-2155 (2 × 10^8^ CFU/mL). Spiked fecal samples were divided into 120 aliquots of 1 mL in Eppendorf tubes and sealed tightly with parafilm, then 30 aliquots each were placed at −70 °C, −20 °C, 4 °C, and room temperature. On days 0, 1, 2, 5, 7, 14, and 28, two 1 mL aliquots were taken from each storage condition and assayed for either vegetative and spore cell counts (Figure 2a) or real-time PCR testing (Figure 2b).

### 4.4. Vegetative and Spore Cell Counts from Plates

For initial vegetative cell counts, 100 µL aliquots of the spiked fecal samples taken from all storage conditions were thoroughly mixed with 900 µL of sterilized PBS. The aliquots were then 10-fold serially diluted with PBS and 100 µL of each dilution was spread onto BHI agar plates. Following incubation at 37 °C under anaerobic conditions for 72 h, the numbers of colonies were enumerated [26,27].

For the counts of spore cells, 200 µL of each spiked fecal sample was mixed with an equal volume of ethanol and incubated at RT for 1 h. The mixtures were then 10-fold serially diluted with PBS and 100 µL of the dilution was spread onto CCFA supplemented with 5% defibrinized horse blood, which was anaerobically incubated and counted as described above [26,27].

### 4.5. Real-Time PCR Detection Assay

Primer 3 (http://bioinfo.ut.ee/primer3-0.4.0/) was used to design *tcdA* (internal fragment of the toxin A gene, CDIF630_00776) and *tcdB* (internal fragment of the toxin B, CDIF630_00773) primers (Table 3). The primer sequences were evaluated by using the Basic Local Alignment Search Tool (BLAST) with specificity of primers. Using all 21 strains (Table A1), we tested the new primers, together with reference primers (Table 3) [28]. The estimated sensitivity of the new qPCR primers (Table A2) was 100% using 21 *C. difficile* strains (Table A1). The new primers detected the two toxin genes at lower cycle quantification (Cq) values than a set of previously published reference primers (Table A2). The limits of detection (LoD) of the new primers for tcdA and tcdB genes were 3.51 × 10^3^ CFU per mL and 1.00 × 10^2^ CFU per mL, corresponding to 35.65 and 34.50 of Cq value, respectively [29].

Total genomic DNA samples were extracted using DNA isolation QIAamp PowerFecal DNA Kit (Qiagen, Hilden, Germany). Concentrations of the extracted DNA samples and their purity were measured using a NanoDrop instrument (Thermo Fisher Scientific, USA). To quantify the total numbers of bacteria, standard curves were generated by plotting Cq values versus the concentrations of purified PCR products obtained via amplification of the genes from the genomic DNA of *C. difficile* ATCC BAA-2155. The real-time PCR reaction (15 μL) contained 1.5 μL of template DNA and 10 μM of each primer, 3 μL nuclease-free water, and 7.5 μL of Faststart Universal SYBR Green Master (Roche, Basel, Switzerland). Real-time PCR amplification reactions were performed with the CFX96 (Bio-Rad, USA) and the following conditions were used: 1 cycle of 94 °C for 3 min; followed by 40 cycles of 94 °C for 30 sec, 60 °C for 30 sec, and 72 °C for 30 sec; 72 °C for 5 min.

### 4.6. Patient and Public Involvement

No patients were involved in the development of the research question, design, or implementation of the study, or interpretation of the results.

## Figures and Tables

**Table 1:** Total numbers of articles on fecal storage conditions published in journals indexed by Google Scholar.

Article Classified in			Number of Articles	Citations	Citations Per Article
Target		Test Method ^1^			
Cell/spore	Viability Cell count	TC TC	7 1	280 92	40.0 92.0
Protein	TcdA/B GDH	CCCNA EIA GDH assay	3 2 2	260 67 5	86.7 33.5 2.5
DNA	*tcdA/B*	NAAT	1	3	3.0
Sum				707	

^1^ Abbreviations: TC, toxigenic culture; CCCNA, cell culture cytotoxicity neutralization assay; GDH, glutamate dehydrogenase; EIA, enzyme immunoassay; NAAT, nucleic acid amplification tests.

**Table 2:** Summary of previous and current studies investigating storage effects on the stool specimens with *C. difficile*.

Experiment	Bowman and Riley (1986) [19]	Weese et al. (2000) [23]	Freeman and Wilcox (2003) [20]	Arroyo et al. (2005) [17]	Alfa et al. (2014) [16]	Becker et al. (2015) [18]	Peterson et al. (2017) [21]	Schora et al. (2018) [22]	This Study
Storage condition									
Period (days) Temp (°C) (An)aerobic Samples	10 0/5/25 Aerobic Patient stool	30~60 4 Both Spiked stool	56 −20/4 Aerobic Spiked stool	56 4/25 Aerobic Patient stool	3 4/RT Aerobic Patient stool	28 4/20 Aerobic Patient stool	60 −20 Aerobic Patient stool	120 −80/−30/4~10 Aerobic Patient stool	28 −70/−20/4/RT Aerobic Spiked stool
Toxigenic culture									
Viability	Recovered during 10 d at 5 °C Few days at 25 °C	14 of 49 isolates recovered after 72 h in aerobic Recovered after 30 d at 4 °C in anaerobic	Recovered during 56 d at −20 °C/4 °C/	Recovered during 56 d at 4 °C/25 °C	Recovered after 72 h at 4 °C/RT	Recovered during 28 d at 4 °C/20 °C	Recovered during at least 60 d at −20 °C or colder (100% agreement between fresh and storage samples)	N/A	Recovered during 28 d at −70 °C/−20 °C/4 °C/RT
Cell counts	N/A	N/A	Stay stable (no storage impact)	N/A	N/A	N/A	N/A	N/A	Decreased until 3 d at −70 °C/−20 °C/4 °C/RT and then stay stable until 28 d
Protein-based									
Cytotoxin assay (CCCNA)	Toxin titer stable at 0 °C Toxin titer decreased (1.4 and 1.7 log) after 2 d at 5 °C/25 °C	N/A	Toxin titer fluctuated during 56 d at 4 °C Toxin titer decreased (5 log) during 56 d at −20 °C	N/A	Toxin effect 100% and 90% reproducible after 72 h at 4 °C/RT	N/A	N/A	N/A	N/A
ELISA assay (EIA)	N/A	Toxin A/B detected for up to 60 d at 4 °C	N/A	N/A	N/A	N/A	N/A	Toxin A/B detected for up to 120 d at −30 °C/−80 °C/4~10 °C	
Antigen assay (GDH)	N/A	N/A	N/A	N/A	GDH remained detectable 100% up to 72 h at 4 °C/RT	GDH remained detectable during 28 d at 4 °C/20 °C	N/A	N/A	N/A
DNA-based									
NAAT	N/A	N/A	N/A	N/A	N/A	N/A	97.6% agreement between fresh and frozen storage samples	N/A	A qPCR-based cell count showed 10^7^–10^8^ CFU/mL at −70 °C/−20 °C/4 °C/RT

N/A, not available.

**Table 3:** Real-time PCR primers and probes specific for toxin A (*tcdA*) and B (*tcdB*) genes for the detection of *C. difficile*.

Primer	Sequence	Target
Designed in this study		
*tcdA*-7582F *tcdA*-7784R *tcdB*-3005F *tcdB*-3161R	CCTGATGGATTTGAATACTTTGC CCATTCGCACCCATAGCTGTA CAGATGCAGCCAAAGTTGTTGA GGGTCACTCGTTTCACTTAGC	*tcdA* *tcdA* *tcdB* *tcdB*
Reference primers used by Kilic et al (2015) [28]		
F R F R	TGATAACGTATAGCTTGACC ATGGTTTACCTCAGATAGG GAAGGATTACCTGTAATTGC CTGCCATTATACCTATCTTAGC	*tcdA* *tcdA* *tcdB* *tcdB*

## Data Availability

https://www.mdpi.com/ethics.

## Appendix A

**Table A1:** Toxigenic strains of *C. difficile* used for analytical sensitivity studies.

ATCC Strain	Strain	Toxinotype	Toxin		
			A	B	CDT
43598 17857 17858 43255 43594 43596 43599 43600 51695 700792 9689 BAA-1382 BAA-1805 BAA-1870 BAA-1871 BAA-1872 BAA1873 BAA-1874 BAA-1875 BAA-2155 BAA-2156	1470 870 1253 VPI 10463 W1194 545 2022 2149 BDMS 18 AN 14797-2 90556-M6S 630 N/A 4118 4111 4206 5283 4205 5325 LBM 0801058 LBM 0801040	VIII O O O O O O O O O O O IIIb IIIb O O O O V XXII O	− + + + + + + + + + + + + + + + + + + + +	+ + + + + + + + + + + + + + + + + + + + +	+ − − − − − − − − − − − + + − − − − − + −

## Appendix B

**Table A2:** Real-time PCR assay used to determine sensitivity using the reference primers and the primers designed in this study based on cycle quantification (Cq).

Name	Toxin A (*tcdA*)			Toxin B (*tcdB*)		
	Ref. ^1^	This Study	Difference	Ref. ^2^	This Study	Difference
ATCC 43598 ATCC 17857 ATCC 17858 ATCC 43255 ATCC 43594 ATCC 43596 ATCC 43599 ATCC 43600 ATCC 51695 ATCC 700792 ATCC 9689 ATCC BAA-1382 ATCC BAA-1805 ATCC BAA-1870 ATCC BAA-1871 ATCC BAA-1872 ATCC BAA1873 ATCC BAA-1874 ATCC BAA-1875 ATCC BAA-2155 ATCC BAA-2156	N/S ^3^ 21.75 21.08 21.76 21.03 23.15 21.47 23.97 20.59 21.36 19.79 20.25 23.67 24.44 21.03 20.65 20.78 22.22 20.20 21.11 21.18	N/S ^3^ 18.48 22.42 18.59 19.12 20.52 19.40 19.46 20.45 19.32 20.14 22.63 18.80 20.47 22.08 21.17 17.11 21.00 20.38 20.42 18.26	N/S ^3^ 3.27 −1.34 3.16 1.91 2.63 2.07 4.50 0.14 2.03 −0.35 −2.38 4.87 3.97 −1.06 −0.52 3.68 1.22 −0.18 0.68 2.92	24.92 26.07 24.73 26.02 25.04 26.92 25.32 28.75 24.19 25.72 24.15 24.40 22.22 28.93 25.17 24.21 19.76 26.11 18.68 25.34 24.93	16.58 16.07 17.12 16.08 18.31 14.72 15.06 14.54 16.57 16.58 18.94 18.74 22.99 17.44 16.64 15.77 18.24 16.52 26.25 15.55 15.80	8.34 10.00 7.61 9.94 6.73 12.20 10.25 14.20 7.62 9.14 5.21 5.65 −0.77 11.50 8.53 8.43 1.52 9.59 −7.56 9.79 9.13
Average	21.58	20.01	1.56	24.83	17.40	7.44

^1^ Ref., F and R for *tcdA*; this study, *tcdA*-7582F and *tcdA*-7784R (please refer to Table 3). ^2^ Ref., F and R for *tcdB*; this study, *tcdB*-3005F and *tcdB*-3161R (please refer to Table 3).^3^ N/S, non-specific.
